# Retinoprotection by BGP-15, a Hydroximic Acid Derivative, in a Type II Diabetic Rat Model Compared to Glibenclamide, Metformin, and Pioglitazone

**DOI:** 10.3390/ijms21062124

**Published:** 2020-03-19

**Authors:** Zita Wachal, Mariann Bombicz, Dániel Priksz, Csaba Hegedűs, Diána Kovács, Adrienn Mónika Szabó, Rita Kiss, József Németh, Béla Juhász, Zoltán Szilvássy, Balázs Varga

**Affiliations:** Department of Pharmacology and Pharmacotherapy, Faculty of Medicine, University of Debrecen, Nagyerdei krt 98., H-4032 Debrecen, Hungary; w.zita@yahoo.com (Z.W.); bombicz.mariann@pharm.unideb.hu (M.B.); priksz.daniel@pharm.unideb.hu (D.P.); csaba.hegedus.1983@gmail.com (C.H.); kovacs.diana@med.unideb.hu (D.K.); szabo.adrienn23@gmail.com (A.M.S.); kiss.rita@med.unideb.hu (R.K.); nemeth.jozsef@med.unideb.hu (J.N.); juhasz.bela@med.unideb.hu (B.J.); szilvassy.zoltan@med.unideb.hu (Z.S.)

**Keywords:** diabetic retinopathy, BGP-15, glibenclamide, metformin, pioglitazone, Goto-Kakizaki rat, electroretinography (ERG)

## Abstract

High blood glucose and the consequential ischemia-reperfusion (I/R) injury damage vessels of the retina, deteriorating its function, which can be clearly visualized by electroretinography (ERG). The aim of the present study was to evaluate the possible retinoprotective effects of systemic BGP-15, an emerging drug candidate, in an insulin resistant animal model, the Goto-Kakizaki rat, and compare these results with well-known anti-diabetics such as glibenclamide, metformin, and pioglitazone, which even led to some novel conclusions about these well-known agents. Experiments were carried out on diseased animal model (Goto-Kakizaki rats). The used methods include weight measurement, glucose-related measurements—like fasting blood sugar analysis, oral glucose tolerance test, hyperinsulinemic euglycemic glucose clamp (HEGC), and calculations of different indices from HEGC results—electroretinography and Western Blot. Beside its apparent insulin sensitization, BGP-15 was also able to counteract the retina-damaging effect of Type II diabetes comparable to the aforementioned anti-diabetics. The mechanism of retinoprotective action may include sirtuin 1 (SIRT1) and matrix metalloproteinase 9 (MMP9) enzymes, as BGP-15 was able to elevate SIRT1 and decrease MMP9 expression in the eye. Based on our results, this emerging hydroximic acid derivative might be a future target of pharmacological developments as a potential drug against the harmful consequences of diabetes, such as diabetic retinopathy.

## 1. Introduction

Diabetes mellitus is a worldwide problem in the developed countries, even more and more people are affected by each year. Based on WHO statistics updated in October 2018 the number of diabetic patients has risen from 108 million in 1980 to 422 million in 2014 [1]. Most chronic complications of diabetes result from macro- and microangiopathy: coronary, cerebrovascular, and peripheral vascular diseases, diabetic nephropathy, neuropathy, and retinopathy.

Diabetic retinopathy is a highly important contributing factor—in many countries the leading cause—of blindness [2]. High blood glucose and the consequential ischemia-reperfusion (I/R) injury damage the vessels of the retina deteriorating its function, a pathological process that can be clearly visualized by the use of electroretinography (ERG), a method which may also be used to measure the disease-ameliorating effects of different research materials [3,4].

**Figure 1:**
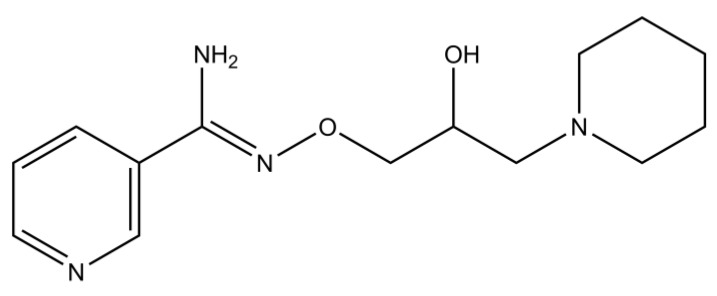
Chemical structure of *O*-(3-piperidino-2-hydroxy-1-propyl)-nicotinic amidoxime, also known as BGP-15, a hydroxamic-acid derivative small molecule (the figure was made using ChemDraw Ultra software ver.12., CambridgeSoft, Perkin Elmer, Waltham, MA, USA).

BGP-15 (Figure 1), a relatively new drug candidate, has been tried out systemically against many different pathologies related with diabetes and ischemia-reperfusion injury, such as heart ischemia-reperfusion injury [5,6], nephrotoxicity [7], neuropathy [8,9], myopathy [10], and most importantly against insulin resistance [11,12]. However, at the time of writing, BGP-15 has not been investigated in diabetic retinopathy, although its mechanisms of action makes it a potential candidate for prevention or treatment of such disorder.

Formerly BGP-15 was demonstrated to induce heat shock protein (HSP) 72 [13], a cytosolic protein belonging to the HSP 60/70 kDa family responsible for e.g., the transport of matrix metalloproteinase 9 (MMP9) into the mitochondria [14]. In diabetes—due to over-transactivation by dysregulated transcription factors (NF-kappaB, PARP1 etc.)—excess levels of MMP9 transported into the mitochondria may lead to mitochondrial damage [15]. The regulation of these aforementioned transcription factors are done by i.a. sirtuin 1 (SIRT1), a nuclear deacetylase enzyme [14,15], which has been linked with diabetic retinopathy: although SIRT1 would promote cell curvival physiologically, it is inhibited in diabetic retinopathy, thus acetylation-deacetylation balance is disturbed [14,16]. Absence of deacetylation increase the binding of different transcription factors (NF-kappaB, PARP1, etc.) to promoter of matrix metalloproteinase 9 (MMP9), which results in transactivation and eventually leads to mitochondrial damage [15] probably in the diabetic retina as well. Mitochondrial damage and eventual death of retinal cells may lead to the deterioration of visual function, a characteristic symtpom of diabetic retinopathy [17]. Whether BGP-15 may affect in this pathway beside HSP72 either SIRT1 or MMP9 and thus the pathogenesis of diabetic retinopathy was still to be discovered.

Based on these former results, the aim of the present study was to evaluate the effects of systemic BGP-15 against the deteriorating effects of glucose on the retina in an insulin resistant animal model, Goto-Kakizaki rat. We also wanted to compare the possible retinoprotective effect of BGP-15 with standard anti-diabetic drugs using electroretinograph. To our best knowledge, such electroretinographical screening of retinal function has not been done yet neither with BGP-15 nor with metformin, glibenclamide or pioglitazon, commonly used representatives of the main anti-diabetic drug-groups. We also attempted to identify a probable action-mechanism for the observed retinoprotection of the used treatments: we wanted to assess any possible effect of BGP-15 on SIRT1 or MMP9 expression.

## 2. Results

### 2.1. Weightgain

**Figure 2:**
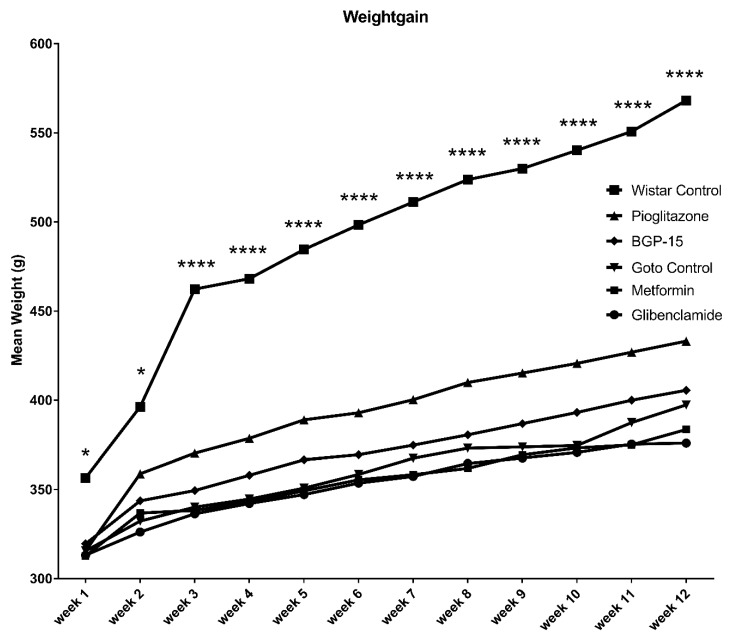
Weight gain of animal groups during the 12 weeks of the experiment. Data is presented as group mean. For a better visibility, standard error of the mean (SEM) of each data points are not plotted. n=6 animals in each group. * *p* < 0.05 compared to all other groups; **** *p* < 0.0001 compared to all other groups. Statistical analysis was done using GraphPad Prism: data was analysed with two-way analysis of variance (ANOVA) test.

**Figure 3:**
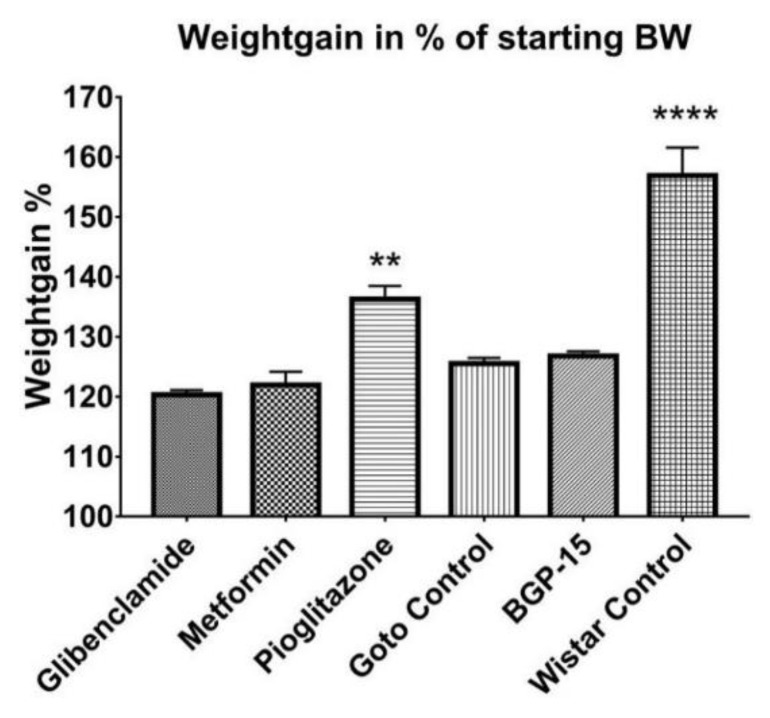
Weight gain percentages of the animal groups: mean weight of animals of each group at the end of the experiment expressed in percentages of their initial mean weight. Data is presented as group mean ± standard error of the mean (SEM); *n* = 6 animals in each group. ** *p* < 0.01 compared to all other groups; **** *p* < 0.0001 compared to all other groups. Statistical analysis was done using GraphPad Prism: after estimation of Gaussian distribution with Shapiro-Wilk normality test data was either analysed with one-way analysis of variance (ANOVA) or non-parametric Kruskal-Wallis test.

Throughout the 12 weeks of the study weight of the animals was measured weekly. A more or less constant increase in bodyweight could be observed in each group (Figure 2). Starting and endpoint mean weights ± SEM of the groups were as follows: Glibenclamide 313.3 ± 4.074–376.0 ± 5.671 g, Metformin 312.8 ± 5.655–383.6 ± 12.268 g, Goto control 315.6 ± 4.070–397.4 ± 6.266 g, BGP-15 319.6 ± 5.566–405.6 ± 9.024 g, Pioglitazone 315.6 ± 3.658–433.2 ± 10.398 g, Wistar control 356.5 ± 3.989–568.2 ± 18.552 g; in all groups week 1 (starting) vs week 12 (endpoint) weights were significantly different (*p* < 0.0001). Weight gain in percentages of starting bodyweight ± SEM of the pioglitazone-treated group (136.3 ± 2.207%) proved to be significantly higher (*p* < 0.01) than the other diseased groups (120.3 ± 0.788%, 121.9 ± 2.228%, 125.5 ± 0.940% and 126.8 ± 0.769% for Glibeclamide, Metformin, Goto control and BGP-15 groups, respectively), while data of healthy Wistar rats (156.9 ± 4.667%) stood out from all the other groups (*p* < 0.0001) as seen in Figure 3.

### 2.2. Fasting Plasma Glucose Results

**Figure 4:**
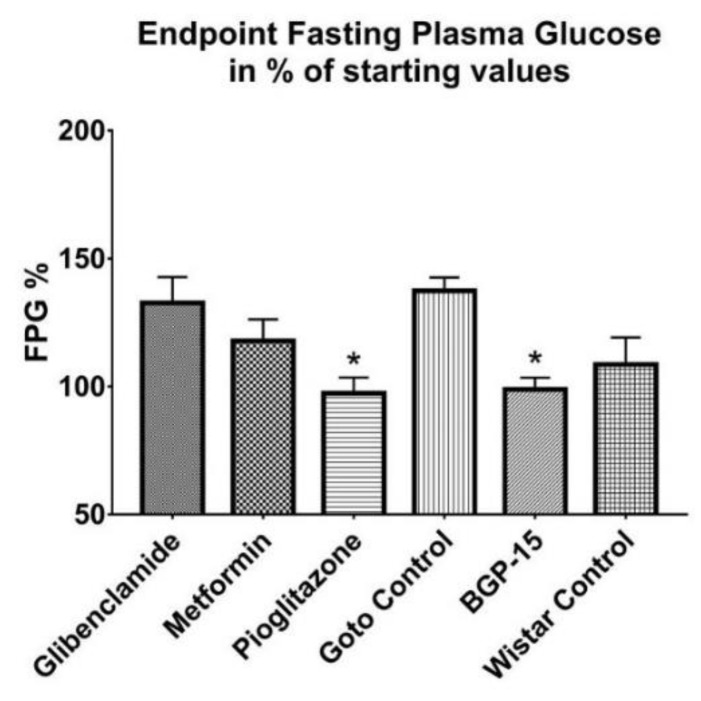
Endpoint fasting plasma glucose values expressed in percentages of starting fasting plasma glucose values. Data is presented as group mean± standard error of the mean (SEM); *n* = 6 animals in each group. * *p* < 0.05 compared to Goto Control group; FPG = fasting plasma glucose. Statistical analysis was done using GraphPad Prism: after estimation of Gaussian distribution with Shapiro-Wilk normality test data was either analysed with one-way analysis of variance (ANOVA) or non-parametric Kruskal-Wallis test.

Throughout the study fasting plasma glucose (FPG) levels of all diseased, Goto-Kakizaki groups revolved around a mean 8–9 mmol/L blood glucose value—without any significant difference between any two groups—while in the meantime Wistar values remained at a significantly lower level, around a mean 5–6 mmol/L (9.2 ± 0.589 mmol/L, 9.4 ± 0.526 mmol/L, 8.2 ± 0.171 mmol/L, 9.2 ± 1.059 mmol/L and 9.4 ± 0.692 mmol/L vs. 5.2 ± 0.178 mmol/L for glibenclamide, Metformin, Pioglitazone, Goto control and BGP-15 vs Wistar control, respectively; *p* < 0.05). In Figure 4 final values of FPG are plotted in percentages of starting values. In case of BGP-15, Pioglitazone and Wistar control groups FPG did not change, endpoint fasting blood sugar in percentage of starting values±SEM turned out to be 97.35 ± 6.116% in pioglitazone-treated group, 98.87 ± 4.532% in BGP-15-treated group and 108.6 ± 10.550% in Wistar group, of which the first two mentioned groups differ statistically significantly (both *p* < 0.05) from Goto control group (137.4 ± 5.219%). Values for glibenclamide- and metformin-treated groups were 132.6 ± 10.15% and 117.8 ± 8.421%, respectively.

### 2.3. OGTT Results

**Figure 5:**
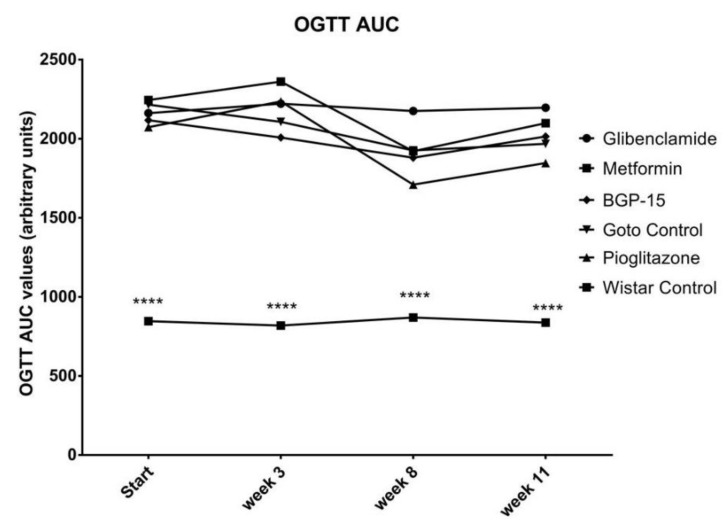
Area Under the Curve (AUC) of blood glucose values measured during Oral Glucose Tolerancy Tests (OGTT). Data is presented as group mean. For a better visibility, standard error of the mean (SEM) of each data points are not plotted. *n* = 6 animals in each group. **** *p* < 0.0001 compared to all other groups. Statistical analysis was done using GraphPad Prism: data was analysed with two-way analysis of variance (ANOVA) test.

**Figure 6:**
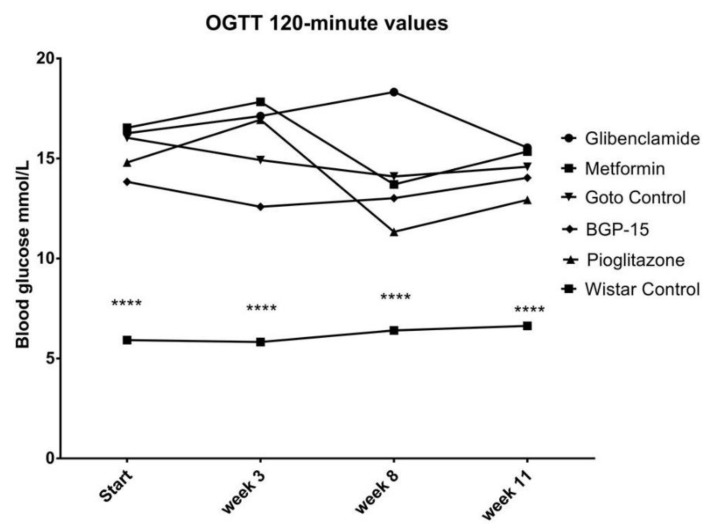
Blood glucose values of the different animal groups at the end of OGTTs (120 min after glucose load). Data is presented as group mean. For a better visibility, standard error of the mean (SEM) of each data points are not plotted. *n* = 6 animals in each group. **** *p* < 0.0001 compared to all other groups. Statistical analysis was done using GraphPad Prism: data was analysed with two-way analysis of variance (ANOVA) test.

Neither Area Under the Curve (AUC) of blood glucose during the Oral Glucose Tolerancy (OGTT), nor 120-min OGTT values showed any difference between the treated groups and the non-treated Goto Control group ( Figure 5; Figure 6). On the other hand, as expected, even the 120-min values of OGTT did not show any signs of diabetes in the healthy Wistar group (Figure 6) as it was the case with the fasting plasma glucose values before: during the study all 120-min blood glucose values of healthy Wistar rats remained under 7.8 mmol/L (5.92 ± 0.073, 5.82 ± 0.183, 6.40 ± 0.148 and 6.63 ± 0.551 mmol/L ± SEM at the start of the study and at week 3, 8 and 11, respectively).

### 2.4. HEGC Results

**Figure 7:**
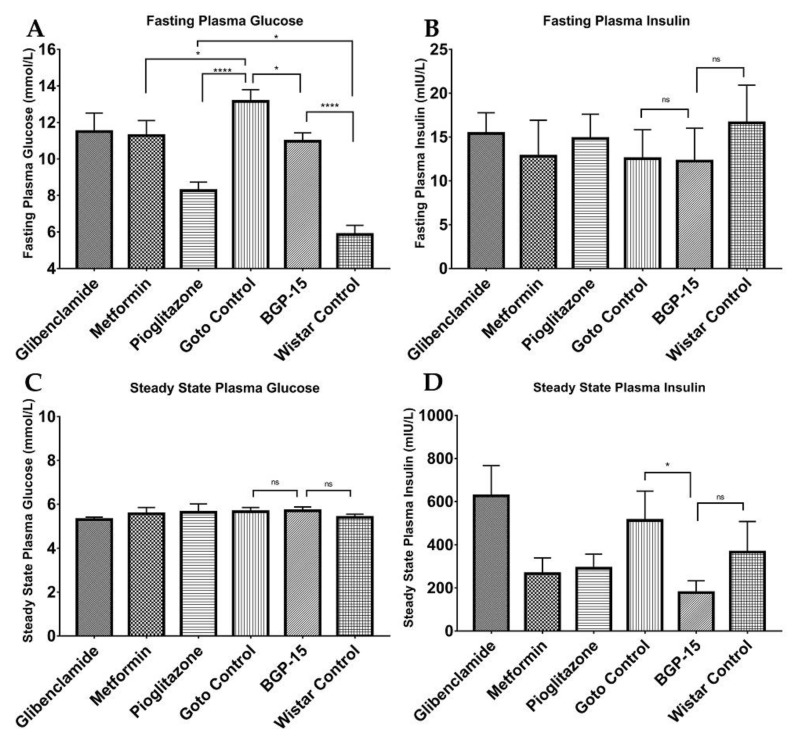
Plasma glucose and insulin values at the start and at the end of hyperinsulinemic euglycemic glucose clamp (HEGC) protocol. (**A**) Fasting plasma glucose values at the start of the HEGC protocol; (**B**) Fasting plasma insulin values at the start of the HEGC protocol; (**C**) Steady state plasma glucose values at the end of the HEGC protocol; (**D**) Steady state plasma insulin values at the end of HEGC protocol. All data are presented as mean ± standard error of the mean (SEM). *n* = 6 animals in each group. * *p* < 0.05; **** *p* < 0.0001; ns = non-significant. Statistical analysis was done using GraphPad Prism: after estimation of Gaussian distribution with Shapiro-Wilk normality test data was either analysed with one-way analysis of variance (ANOVA) or non-parametric Kruskal-Wallis test.

As shown in Figure 7, there were significant differences between plasma insulin levels at the end of the hyperinsulinemic euglycemic glucose clamp (HEGC) method: while healthy Wistar rats presented a 366.6 ± 141.3 mU/L mean ± SEM value, mean value of BGP-15-treated Goto-Kakizaki rats turned out to be 178.3 ± 54.71 mU/L that differed significantly from the 513.0 ± 135.9 mU/L value of Goto control group (* *p* < 0.05). Glibenclamid failed to present amelioration of insulin resistance as seen from the relatively high insulin levels (626.3 ± 140.7 mU/L) at the end of HEGC protocol, but both metformin and pioglitazone produced an insulin-senzitizing effect (with values of 266.6 ± 72.93 and 291.5 ± 65.46 mU/L, respectively). If we look at the direct measures of fasting plasma glucose values (Figure 7A), it is conspicuous that not only metformin (11.28 ± 0.822; *p* < 0.05 vs. Goto control (13.15 ± 0.650 mmol/L)) and pioglitazon (8.27 ± 0.463 mmol/L; *p* < 0.0001 vs. Goto control), but also BGP-15 (10.97 ± 0.460 mmol/L) was able to lower blood glucose significantly (*p* < 0.05 vs. Goto control), although this decrease was not as pronounced as with pioglitazone. However, even pioglitazone was not able to reach the level of healthy animals (*p* < 0.05 in comparison of pioglitazone vs. Wistar control (5.86 ± 0.506 mmol/L)). Fasting plasma blood glucose value ± SEM for glibenclamide-treated group was 11.5 ± 1.016. There were no significant differences between the groups in case of fasting plasma insulin and steady state blood glucose.

**Figure 8:**
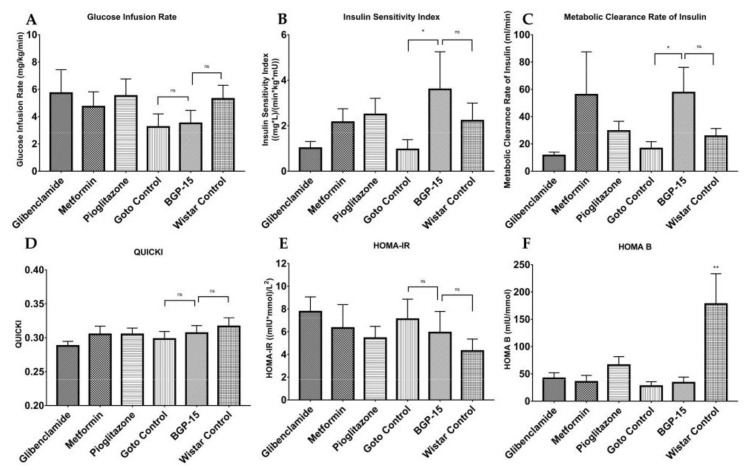
Additional calculated indices based on HEGC protocol. **A**: Glucose Infusion Rate; **B**: Insulin Sensitivity Index; **C** Metabolic Clearance Rate of Insulin; **D**: Quantitative insulin sensitivity check index (QUICKI); **E**: Homeostasis model assessment of insulin resistance (HOMA-IR); **F**: homeostasis model assessment of B-cell function (HOMA-B). All data are presented as mean ± standard error of the mean (SEM). *n* = 6 animals in each group. * *p* < 0.05; ns = non-significant; In panel **F** ** *p* < 0.01 compared to all other groups. Statistical analysis was done using GraphPad Prism: after estimation of Gaussian distribution with Shapiro-Wilk normality test data was either analysed with one-way analysis of variance (ANOVA) or non-parametric Kruskal-Wallis test.

Further analyses of direct HEGC values granted us calculated indices (Figure 8), such as the glucose infusion rate (GIR), Insulin Sensitivity Index (ISI), metabolic clearance rate of insulin (MCRI), quantitative insulin sensitivity check index (QUICKI), homeostasis model assessment of insulin resistance (HOMA-IR), and homeostasis model assessment of B-cell function (HOMA-B). As seen from ISI and MCRI graphs BGP-15 values ± SEM (3.596 ± 1.656 and 57.57 ± 18.500, respectively) differed significantly from Goto control values ± SEM (0.956 ± 0.432 and 16.57 ± 5.095, respectively), and at the same time there were no difference compared to healthy control values (2.22 ± 0.776 and 25.67 ± 5.650, respectively), even slightly (but statistically non-significantly) higher values are seen. In the other diagrams, differences did not reach the level of statistical significance (with respect of reasonable comparisons).

### 2.5. Electroretinographical Results

**Figure 9:**
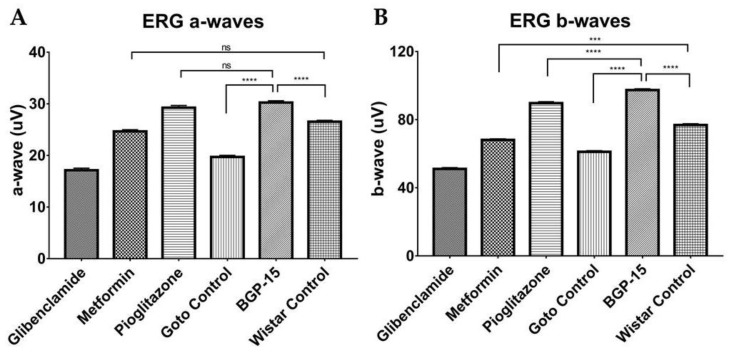
Results of electroretinographical (ERG) measurements. (**A**) Group means of a-waves; (**B**) Group means of b-waves. All data are presented as group means± standard error of the mean (SEM). *n* = 6 animals in each group. In both inserts all unmarked comparisons (between any two groups) are **** *p* < 0.0001. *** *p* < 0.001; ns = non-significant. Statistical analysis was done using GraphPad Prism: after estimation of Gaussian distribution with Shapiro-Wilk normality test data was either analysed with one-way analysis of variance (ANOVA) or non-parametric Kruskal-Wallis test.

According to ERG measurements, group-trends in a- and b-waves were very similar (compare Figure 9A vs. 9B). BGP proved to produce higher a- and b-wave amplitudes (30.25 ± 0.342 µV and 97.39 ± 0.708 µV, respectively), than what could be observed in Goto control group (19.7 ± 0.315 µV and 61.11 ± 0.672 µV, respectively). Moreover, BGP-values were statistically significantly higher than healthy Wistar values (26.54 ± 0.267 µV and 76.83 ± 0.767 µV, mean a- and b-wave amplitudes, respectively). This effect of BGP-15 treatment on a- and b-wave amplitudes was similar or better than metformin- and pioglitazone treatment (metformin mean a- and b-waves: 24.66 ± 0.316 µV and 68.09 ± 0.628 µV; pioglitazone mean a- and b-waves: 29.25 ± 0.426 µV and 89.68 ± 0.862 µV, respectively). The former mentioned three treatments that proved to provide similar or higher amplitudes than healthy Wistar animals.

### 2.6. Western Blot Results

**Figure 10:**
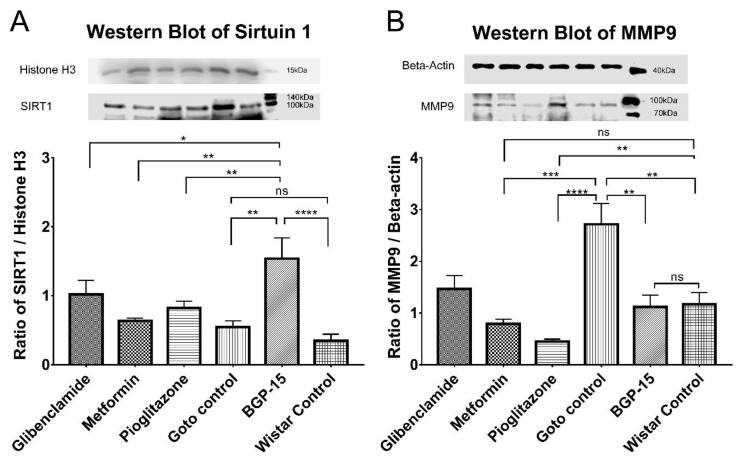
Western Blot results. (**A**) Expression levels of Sirtuin 1 (SIRT1) in the different groups of the study. (**B**) Expression levels of Matrix metalloproteinase 9 (MMP9) in the different groups. Detected proteins: Histone H3 (~17kDa), SIRT1 (~120kDa), beta-actin (~42kDa), MMP9 (~92kDa). All data are presented as group mean ± standard error of the mean (SEM); *n* = 6 animals in each group. * *p* < 0.05; ** *p* < 0.01; *** *p* < 0.001; **** *p* < 0.0001; ns = non-significant. Statistical analysis was done using GraphPad Prism: after estimation of Gaussian distribution with Shapiro-Wilk normality test data was either analysed with one-way analysis of variance (ANOVA) or non-parametric Kruskal-Wallis test.

Primary antibodies against sirtuin 1 and matrix metalloproteinase 9 were used on isolated proteins after they were separated using sodium-dodecyl-sulphate polyacrylamide gel electrophoresis (SDS-PAGE) and electrophoretically transferred onto nitrocellulose membranes. As seen in Figure 10 significant differences were observable in case of the aforementioned two proteins in the BGP-treated group.

Sirtuin 1 expression (Figure 10A) was boosted by BGP-15 treatment: standardized-normalized pixel density of BGP-15 treated group proved to be significantly higher than that of both Goto control and Wistar control groups (1.539 ± 0.301 vs 0.6463 ± 0.094 and0 0.2346 ± 0.083, *p* < 0.01 and *p* < 0.0001, respectively). Statistically there was no significant difference between Goto control and Wistar control groups. BGP-15 value was observed to be significantly higher than values of all other treatment groups (1.020 ± 0.205, 0.6371 ± 0.040 and 0.8204 ± 0.1015 of glibenclamide-, metformin- and pioglitazone-group, *p* < 0.05, *p* < 0.01 and *p* < 0.01, respectively). In glibenclamide- and pioglitazone-treated groups SIRT1 level turned out to be significantly higher than in Wistar control group (*p* < 0.05 in both comparisons, not marked in figure).

Expression of MMP9 (Figure 10B) was significantly elevated in the diabetic Goto control group as compared to the healthy Wistar control group (2.716 ± 0.402 vs 1.167 ± 0.229 *p* < 0.05). This increase was ameliorated by all treatment groups (BGP-15, glibenclamide, metformin and pioglitazone; 1.1210 ± 0.225, 1.466 ± 0.257, 0.7875 ± 0.093 and 0.4484 ± 0.047, respectively). The decrease in MMP9 by BGP-15 compared to Goto control group was significant (*p* < 0.05) so that mean value of BGP was similar to Wistar control group: there were no significant difference between the latter two groups. Glibenclamide managed to perform the same as BGP: its value differed significantly from Goto control (*p* < 0.05), but not from Wistar control (comparisons not marked in figure). Metformin and pioglitazone were even more effective (*p* < 0.05 in metformin vs Wistar control and *p* < 0.01 in pioglitazone vs Wistar control comparison).

Based on the specifications of the manufacturer of the MMP9 antibody, the antibody may detect other forms of MMP9 as well, as “Processing of the precursor [of MMP9] yields different active forms of 64, 67 and 82 kDa” (Abcam). Other bands with lower molecular weight were indeed observable on the blots of MMP9, and were also analysed; however, there were no statistical differences between the bands of any two groups (Supplementary Figure S1).

## 3. Discussion

As the incidence of diabetes mellitus is increasing so is the prevalence of diabetic retinopathy, a probable cause of blindness. Indeed, diabetic retinopathy has become the leading cause of blindness in many developed countries [18].

In the present study, we wanted to assess the possible retinoprotective effects of BGP-15, an emerging hydroximic acid derivative, in a diabetic setting, and compare it to well-known anti-diabetics such as glibenclamide, metformin and pioglitazone.

Goto-Kakizaki (Goto) rat is one of the spontaneous diabetic, non-obese animal models for the research of diabetes mellitus Type II and its consequences, such as retinopathy. Thus, in our experiment, Goto rats showed much less pronounced increase in weight compared to their breeding control, Wistar rats, the latter producing significant weight gain during the study period (Figure 2 and Figure 3). There were significant differences, however, between the Goto groups: pioglitazone, although being a widely used anti-diabetic agent, seemed to make the rats prone to obesity. Based on the scientific literature this is a rather controversial finding, as pioglitazone is emerging as a treatment choice for nonalcoholic steatohepatitis (NASH) in both diabetic and nondiabetic patients [19], although other sources also mention weight gain as a possible adverse effect of the drug [20,21]. BGP-15 treatment did not differ significantly from the other Goto groups regarding weight gain during our study.

Blood glucose values (Figure 4) were only measured as part of the usual protocol of any diabetic study, although it bore no novel information about any of the used drugs. Some fluctuation could be observed, which according to the scientific literature and data provided by the distributor of the animal model (Charles River) is normal [22,23]. Although blood glucose-change due to BGP-15 treatment during the whole study was significantly low, BGP did not achieved such low endpoint fasting plasma glucose values as e.g., pioglitazone, but was comparable with metformin, a first-line treatment in Type II diabetes mellitus [24].

Similarly, OGTT results (Figure 5 and Figure 6) confirmed our used animal model—according to World Health Organization, any 120-min OGTT result above 11,1 mmol/liter means diabetes mellitus [25]—however this was the first time to demonstrate the effect of BGP-15 on OGTT to be comparable to well-known anti-diabetic agents. Results of the latter agents can be compared to the work of others: similar measurements can be found in the scientific literature for metformin, pioglitazone, and gliclazide, another sulfonylurea, on human patients with Type 2 diabetes [26].

During the HEGC protocol (Figure 7 and Figure 8), in case of steady state plasma insulin levels, BGP-15 could reach a significantly low value compared to the Goto control group: the less insulin is needed to maintain a constant plasma glucose level around 5.5 mmol/L, the less insulin resistant an animal is [27]. This is fully consistent with the calculated indices such as Insulin Sensitivity Index. Our results demonstrate for the first time that a 12-week-long BGP-15 treatment is able to enhance insulin sensitivity in spontaneously diabetic rats comparable to well-known anti-diabetics such as glibenclamide, metformin and pioglitazone. Results of the latter agents are similar to those found in the scientific literature [28,29].

An important novelty in our present article is the ability of BGP-15 demonstrated to be able to counteract the retinal function-deteriorating effect of type II diabetes in spontaneously diabetic Goto-Kakizaki rats (Figure 9), an effect that was comparable to pioglitazone- and metformin treatment. As a comparison to scientific literature: electroretinographical experiments were carried out formerly on high-fat diet-induced diabetic mouse model, although, researchers could not verify retinoprotection on mice elicited by metformin treatment [30]. This was not the case with pioglitazone, which has been proven to prevent glial cell apoptosis in a glaucoma rat model, in which ischemia-reperfusion was induced artificially by means of high intraocular pressure: here pioglitazone attenuated the decrease of ERG and VEP wave amplitudes caused by I/R [31]. Glibenclamide has been tried out together with ischaemic preconditioning, a method that is known to attenuate ischaemic damages to retina: in this experiment glibenclamide was considerably more effective injected intraocularly prior to preconditioning [32]. Similarly, in our present experiment, all treatments were commenced before the onset of diabetic ischaemia-development, the microcirculation-deteriorating effect of diabetes, which causes almost all diagnostic symptoms of diabetic retinopathy [33,34]. Such preventive measures with a future BGP-15 treatment—by means of administration of this retinoprotective medication to diabetes-prone patients—could counteract development of diabetic visual disturbances due to diabetic retinopathy.

In our experiment, BGP-15 and pioglitazone were able to achieve even higher electroretinographical waves than healthy Wistar rats, which was a suprising result, but not impossible. A possible reason to smaller ERG wave amplitudes is a deeper general anesthesia [35], which might be the cause in case of healthy Wistars: due to their higher bodyweight, Wistar rats did get much higher doses of general anaesthetic (ketamin/xylazine 100/10 mg/kg bodyweight), and we observed that—probably due to the larger fat reservoirs—these animals woke up from their sleep later than GK rats. In addition, there is a slight chance that BGP-15 or pioglitazone may senzitize the retina to light with an unknown mechanism. This possibility sets direction for future scientific research.

Action-mechanism pathways behind the retinoprotective effects of BGP-15 were outlined by our Western Blot results (Figure 10): BGP-15 is able to modify the expression of sirtuin 1 and matrix metalloproteinase 9 enzymes to alleviate the symptoms of diabetic retinopathy. These two enzymes share a common pathway based on former scientific research: SIRT1 deacetylates and thus inhibits protein complexes [16]—comprising proteins such as poly-(ADP-ribose)-polymerase 1 (PARP1), nuclear factor kappa B (NFκB) and activator protein 1 (AP1)—that would initiate transcription of i.a. MMP9 in the retina [36]. However, in diabetes both the expression and the activity of SIRT1 are inhibited, thus by inhibition of inhibition, the above-mentioned transcription factors promote the synthesis of MMP9 [14,15]. This leads to excessive transactivation and consequential accumulation of MMP9 even in mitochondria damaging the mitochondrial membrane and releasing cytochrome c into the cytosol thereby activating apoptosis [37].

In our study, SIRT1 levels of Wistar proved to be unusually low, even seemingly lower than that of Goto control group, although the difference did not reach the level of statistical significance. According to other scientific articles, calorie restriction increases SIRT1 concentrations [38] while in contrast overweight and obesity is associated with SIRT1 down-regulation [39,40], which might have been the reason in our experiment for the lower than usual SIRT1 expression in case of the Wistar rats that produced significant weight gain during the study.

According to our present results, BGP-15 may counteract the abovementioned mitochondria damaging events as it is able to increase the expression of SIRT1 significantly, even more than other anti-diabetics such as glibenclamide, metformin or pioglitazone. For easy comparison with the scientific literature: based on former results metformin is known to elevate the level of SIRT1 in the retina, by which it contributes to resistance against cellular metabolic memory of retinal cells induced by high glucose [41]. In case of glibenclamid, however, to best of our knowledge this is the first time to demonstrate SIRT1-elevating effect in the eye, although in this matter hypotheses could be drawn from other studies involving non-retinal tissues [42]. Consistent with former results in kidneys [43,44] and liver [45], pioglitazone treatment in our study produced elevation in SIRT 1 expression in the diabetic eye as well, which is also a novel result.

At the same time, we demonstrated here for the first time that BGP-15 is able to decrease the expression of MMP9 to the level of the healthy control. Formerly glibenclamide has been shown to decrease MMP9 levels but only in metastatic breast cancer [46] and brain [47]. Our result regarding the MMP9-lowering effect of glibenclamide in the diabetic eye is a novelty. Metformin was proved to reduce MMP9 synthesis in many different pathologies e.g., in adipocytes in insulin resistant diabetes [48], in breast cancer [49], in spinal cord injury [50], etc., but so far no scientific article demonstrated this effect in eye of diabetic animals as in this present study. The same applies to pioglitazone: pioglitazone was demonstrated to lower MMP9 levels in e.g., murine peritoneal macrophages [51], in lung [52] and breast [53] cancer cells, as well as in serum of atherosclerotic rabbit model [54]. However, there is no mention in the scientific literature yet that the agent decreases MMP9 expression in the eye. Therefore, in addition to demonstrating the efficacy of BGP-15, we demonstrated novel facts from three well-known anti-diabetics that could have been inferred from previous results, but no one has described yet in diabetic eye.

A most recent article about BGP-15 from another research group [55] came out to conclude that BGP protects the mitochondria, which is fully consistent with our results presented here. Although in the aforementioned article BGP was applied in acetaminophen-induced hepatotoxicity and the action-mechanism pathway (prevention of JNK activation and consequential lowering of autophagy markers) also differs from ours, but there might be a possible connection between our results and theirs as follows.

In diabetes a subclinical ischaemia is suggested to have a possible, important role in retinal neurodegeneration, furthermore this neurodegeneration is also present without developed diabetic retinopathy and even in patients with good metabolic control [56]. Oxidative stress alone is able to trigger the c-Jun amino-terminal kinase (JNK) mitogen-activated protein (MAP) kinase pathway, which—by enhancement of mitochondrial outer membrane permabilization (MOMP) [57] and formation of mitochondrial permeability transition pores (MPTP) [58]—leads to the damage of the mitochondrial membrane letting out proapoptotic mediators and perhaps MMP9. This latter enzyme seems to be of major importance in the development of diabetic retinopathy, while damage of retinal mitochondria [59], and oxidative stress are both proven to be present in the diabetic retina [60], thus the two pathways must cross-talk with each-other.

Indeed MMP9 can be the link: while in diabetes MMP9 is overexpressed due to SIRT1-deficit [14,15], there are also results confirming activation of MMP9 in the diabetic retina by extracellular signal-regulated kinase (ERK) MAP kinase pathway [37,61]. Thus the accumulated and activated MMP9 then damages the mitochondrial membrane [37], which is the same result as in the case of JNK MAP kinase pathway, which BGP-15 is able to inhibit. However, in the present experiment, activity of MMP9 was not measured, which is a limitation of the study. Further experiments are needed to assess any possible interactions of BGP with the ERK MAP kinase pathway as well, whether the agent is able to inhibit MMP9-activation.

All things considered, if we want to assess all our present results, we can conclude that BGP-15, pioglitazone and metformin do not share the exact same mechanism of action. Although the effect of pioglitazone in increasing SIRT1 is only a non-significant trend, and metformin does not increase it at all, in contrast, both substances showed a much more potent MMP9-decreasing effect than BGP-15. Nevertheless, although metformin does decrease MMP9 levels, it is still inferior to pioglitazone and BGP-15 functionally. Metformin’s inability to powerfully decrease blood sugar in Goto-Kakizaki rats in our study may be related to its lesser efficacy in function as well, although it still increased retinal function based on ERG measurements and it was good at decreasing MMP9. In contrast the effect of pioglitazone is corroborated by its blood glucose-decreasing effect. In case of BGP-15, blood glucose lowering is unlikely to contribute to its functional effect, although its glucose lowering effect was comparable to that of metformin. It is obvious that these agents are differently involved in the different mechanisms of action, thus have different pathways to act through: Glibenclamide failed to provide any significance in any of the abovementioned comparisons; metformin was best at decreasing MMP9 and was slightly good in increasing function and decreasing blood glucose but did not alter SIRT1; pioglitazone was demonstrated to be best in MMP9-decreasing and blood glucose-decreasing and in increasing retinal function but not in increasing SIRT1; BGP-15 proved to be the best in our experiment in increasing SIRT1 and retinal function, and was good in decreasing blood sugar and MMP9.

## 4. Materials and Methods

### 4.1. Animals and Groups

Male Goto-Kakizaki and Wistar rats (10-week old; 250–300g) were purchased for the study from Charles River Laboratories International, Inc. (Wilmington, MA, USA). The Goto-Kakizaki (GK) model—according to Charles River—is a non-obese Wistar substrain, which develops Type 2 diabetes; its control is the Wistar rat (Crl:WI). Animals were caged and cared for in compliance with international regulations on animal research (ARVO (Statement for the Use of Animals in Ophthalmic and Vision Research) and the NIH guidelines). All methods used during the study were approved by Institutional Animal Care Committee of University of Debrecen, Hungary (18/2013/DE MÁB). Animals had free access to water and were fed standard rodent chow ad libitum.

After two weeks of acclimatization animals were randomly divided into the following groups (*n* = 6 in each group): a Goto-Kakizaki control group (untreated, diseased), a Wistar control groups (untreated, healthy) and four treatment groups involving Goto-Kakizaki rats: BGP-15, metformin, glibenclamide and pioglitazone. The treatments were gavaged through an oro-gastric stainless steel feeding tube daily in the following dosages: BGP-15 10 mg/kg, metformin 100 mg/kg, glibenclamide 5 mg/kg and pioglitazone 10 mg/kg. All treatment agents were obtained from Sigma-Aldrich-Merck KGaA (Darmstadt, Germany). The two control groups received solvent gavaged. Doses were chosen based on former research [61,62,63,64,65]. Weight of the animals were measured once weekly for the 12 weeks of the study.

### 4.2. Glucose-Related Measurements

Fasting blood sugar analyses were carried out at the start of the treatment period, then at the start of week 3, 8, 11, and right before the termination of the study (“end” timepoint). An oral glucose tolerance test (OGTT) was also carried out in all aforementioned timepoints except at the end. At the end of the experiment all animals undergone hyperinsulinemic euglycemic glucose clamp (HEGC) to assess their insulin sensitivity or insulin resistance. Each of the formerly mentioned methods were carried out after an overnight fast.

#### 4.2.1. Fasting Blood Glucose

For fasting, blood sugar analyses blood was collected from tail vein, then blood glucose concentration was calculated by an Accu-Check glucose meter (Roche Diagnostics, Mannheim, Germany).

#### 4.2.2. Oral Glucose Tolerance Test (OGTT)

For OGTT measurements animals were given 2 g/kg glucose (Sigma-Aldrich-Merck KGaA, Darmstadt, Germany) through an oro-gastric feeding tube (gavage) and sugar level of tail blood was analysed after 15, 30, 60, 90 and 120 min. As baseline values fasting blood sugar levels were used (OGTT 0-min timepoint). During the evaluation of OGTT results the following equation was used to calculate Area Under the Curve (AUC), where ‘*n*’ is the number of measuring timepoints:

(1) $$AUC=\left(\left(\frac{c1+c2}{2}\right)\times \left(t2-t1\right)+\ldots +\left(\frac{cn1+cn}{2}\right)\times \left(tn-tn1\right)\right)$$

#### 4.2.3. Hyperinsulinemic Euglycemic Glucose Clamp (HEGC)

At the end of the experiment after the fasting glucose analysis, all animals were anaesthetized by ketamin/xylazine mixture (100/10 mg/ttkg; ketamin from Calypsol, Richter Gedeon Ltd., Budapest, Hungary; xylazine from Nerfasin, Le Vet B.V., Oudewater, Netherlands) and HEGC protocol was carried out similar to a formerly used protocol [62], as follows. First, trachea was surgically revealed and a canule was inserted. Then two jugular veins were cannulated for administration of glucose solution (20%) and insulin (6 mIU/min (milli international unit per minute); Humulin R, Eli Lily, Indianapolis, IN, USA), respectively, and a carotid artery for taking blood from the animal. Blood sugar values were measured with an Accu-Check glucometer from blood samples taken every 10 min. Euglycemia was maintained by means of adjusting the rate of glucose infusion (GI). Steady state GI was registered when the blood glucose level stabilized for at least 20 min. Blood samples for plasma insulin measurement were taken from the carotid artery at the start and at the end of HEGC protocol. Insulin measurements were carried out from plasma from blood samples centrifuged for 2 min at 4 °C and 10,000 *g* (Centrifuge 5415R, Eppendorf GmbH, Hamburg, Germany). After the HEGC protocol, all animals were gently exterminated by overdosing the anaesthetic ketamin/xylazine combination. Thereafter, eyes of the animals were removed for further microbiological analysis.

#### 4.2.4. Calculated Indices

From the results of direct measurements before and during the HEGC protocol—i.e., body weight (BW), fasting plasma glucose (FPG), fasting plasma insulin (FPI), rate of glucose infusion (GI), rate of insulin infusion (II), steady state plasma glucose (SSPG), steady state plasma insulin (SSPI)—different indices were calculated [66], the formulas of which are as follows:

GIR = glucose infusion rate (not to be mistaken with the rate of the glucose infusion (GI))

(2) $$GIR=\frac{GI\;\left(µL/min\right)}{BW\;\left(g\right)}*220$$

The used correcting factor (×220) is due to concentration of the glucose infusion, which was 20% (220 g glucose in 1000 mL of water; Glucose TEVA 20% Solution, Teva Ltd., Debrecen, Hungary), so that the final unit of GIR will be: mg/min/kg.

ISI = Insulin Sensitivity Index; unit: (mg × L)/(min × kg × mIU)

(3) $$ISI=\frac{GIR\;(mg/min/kg)}{SSPI\;\left(mIU/L\right)}$$

MCRI = Metabolic Clearance Rate of Insulin; unit: mL/min

(4) $$MCRI=\frac{II\;\left(mIU/min\right)}{SSPI\;\left(µIU/mL\right)-FPI\left(µIU/mL\right)\;}*1000$$

The correcting factor (×1000) was used due to the conversion of the milli international unit (IU) into micro IU values.

QUICKI = QUantitative Insulin sensitivity ChecK Index (without unit of measure)

(5) $$QUICKI=\frac{1}{log\;FPI\left(µIU/mL\right)+\left(logFPG\left(mg/dL\right)\times 18\right)}$$

The correcting factor (×18) was used because of the conversion of mmol/L into mg/dL.

We used homeostatic model of assessment (HOMA) for calculating insulin resistency (HOMA-IR) based on the work of Matthews et al. [67]

(6) $$HOMA-IR=\frac{FPI\left(µIU/mL\right)*FPG\left(mmol/L\right)}{22.5}\;$$

As the µIU/mL = mIU/L, the final unit of HOMA-IR will be: (mIU × mmol)/L^2^.

To assess the pancreas β-cell function, we used HOMA-B calculation method, based on the same article [67].

(7) $$HOMA-B=\frac{20*FPI\left(µIU/mL\right)}{FPG\left(mmol/L\right)-3.5}$$

As the µIU/mL = mIU/L, the final unit of HOMA-B will be: mIU/mmol.

### 4.3. Eletroretinography

One day before the extermination of the animals electroretinographical assessments were carried out according to a formerly used method [3] as follows. The animals were anaesthetized by a mixture of ketamin/xylazine (100/10 mg/kg), followed by the administration of cyclopentolate for pupil dilation (Humapent, Teva Ltd., Debrecen, Hungary), one drop in each eye. After 20 min of dark adaptation, scotopic electroretinogram was registered using five needle electrodes. Two reference electrodes were put in each earlobes, two measuring electrodes were inserted carefully into the surface of the cornea without perforating it, and finally one electrode served as common earth, pierced into the skin of the glabella. To prevent the eyes from dry-out, a carbomer-based eye gel (Vidisic, Bausch&Lomb, Berlin, Germany) was used, which also enhanced electrical contact of the cornea and the electrodes. Based on the guidelines of the International Society for Clinical Electrophysiology of Vision (ISCEV) electroretinographical measurements were carried out in darkness while eyes were illuminated with a stroboscope (20 cd/m^2^, 0.5 Hz) [68]. The electrical signals of the retina generated by light flashes were led to a computer through an amplifier and an analog-to-digital converter (Bridge Amp and PowerLab, ADInstruments, Sydney, Australia) to be analysed with PowerLab Chart software (Version 5.2.2, ADInstruments, Sydney, Australia).

### 4.4. Western Blot

Right after the extermination of the animals, eyes were extracted and put on ice for further molecular biological examinations. Whole eye samples were homogenized by a disperser (IKA-WERKE, Staufen, Germany) then proteins were isolated using a buffer containing 25 mM Tris, 25 mM NaCl, 0.5 mM EDTA, protease inhibitor cocktail and distilled water (all from Sigma-Aldrich-Merck KGaA, Darmstadt, Germany). After centrifugation at 10,000 rpm for 20 min at 4 °C the cytosolic proteins were aspirated together with the supernatant, while nuclear proteins were further incubated for 1 h with the same buffer supplemented by Triton X 100, a tenside (Sigma-Aldrich-Merck KGaA, Darmstadt, Germany), followed by another centrifugation (14000 rpm, 10 min, 4 °C), after which the nuclear proteins were aspirated together with the supernatant. From a small portion of the supernatants total protein concentration was measured with a spectrophotometer (FLUOstar Optima, BMG Labtech, Ortenberg, Germany), the rest was mixed with Laemmli Sample buffer (Sigma-Aldrich-Merck KGaA, Darmstadt, Germany) and was separated using SDS-Polyacrylamide gel electrophoresis (12% gel, 40 mA for 100–120 min). Proteins were then transferred to a nitrocellulose membrane (GE Healthcare, Darmstadt, Germany), blocked in 3% BSA-solution (Sigma-Aldrich-Merck KGaA, Darmstadt, Germany) and incubated overnight with primary anti-bodies (anti-SIRT1 mouse monoclonal antibody, detecting SIRT1 (~110kDa), Cat#ab110304; Abcam, Cambridge, UK; anti-MMP9 rabbit polyclonal antibody, detecting MMP9 (~92kDa), Cat#ab38898, Abcam, Cambridge, UK; anti-HistoneH3 recombinant rabbit monoclonal antibody, detecting Histone H3 (~17kDa) Cat#701517 ThermoFisher Scientific, Waltham, MA, USA; anti-beta-actin mouse monoclonal antibody, detecting beta-actin (~42kDa), Cat#A5316, Sigma-Aldrich-Merck KGaA, Darmstadt, Germany). The next day, after washing the membranes with distilled water, secondary anti-bodies conjugated with horseradish-peroxidase enzyme were applied to the membranes (anti-mouse antibody Cat#A4416; anti-rabbit antibody Cat#A0545; both from Sigma-Aldrich-Merck KGaA, Darmstadt, Germany), after which blots were made visible using a LiCor C-Digit^®^ blot scanner (LI-COR Inc., Lincoln, NE, USA) and WesternBright™ enhanced chemiluminescent substrate (Advansta Inc., Menlo Park, CA, USA). After normalization to the background and standardization to a housekeeping protein (HistoneH3 or beta-actin), scanned images of three blots of any treatment groups were analysed with ImageJ software (version 1.51, National Institutes of Health, Bethesda, MD, USA).

### 4.5. Statistical Analyses

All acquired data were statistically analysed with the help of GraphPad Prism software (version 7.0, GraphPad Software Inc., La Jolla, CA, USA). After estimation of Gaussian distribution with Shapiro-Wilk normality test data was either analysed with one-way analysis of variance (ANOVA) or non-parametric Kruskal-Wallis test. In case of analysing group values in different timepoints, two-way analysis of variance was used. A comparison was considered significant, if probability values were lower than 0.05. The level of significance was indicated as follows: * *p* < 0.05; ** *p* < 0.01; *** *p* < 0.001; and **** *p* < 0.0001. All data are presented as mean ± standard error of the mean (SEM).

## 5. Conclusions

In conclusion, by elevating the mitochondria-defender SIRT1 expression and at the same time decreasing the mitochondria-destroyer MMP9 expression in the diabetic eye, BGP-15 is able to counteract the retinal function-deteriorating effect of diabetes. Based on our results this emerging hydroximic acid derivative might become a future target of pharmacological developments as a potential drug against the harmful consequences of diabetes, such as diabetic retinopathy.

## Supplementary Materials

Supplementary materials can be found at https://www.mdpi.com/1422-0067/21/6/2124/s1.
